# Evaluating AI models for food and alcohol advertisement classification against human benchmarks

**DOI:** 10.1038/s41598-026-42426-x

**Published:** 2026-03-11

**Authors:** Paula-Alexandra Gitu, Roberto Cerina, Alexander Grigoriev, Stefanie Vandevijvere

**Affiliations:** 1 Department of Data Analytics and Digitalization, Maastricht University, Maastricht, 6211 LM the Netherlands; 2 Institute for Logic, Language and Computation, University of Amsterdam, Amsterdam, 1098 XG the Netherlands; 3 Epidemiology and Public Health, Sciensano, Bruxelles, 1050 Belgium

**Keywords:** Health care, Mathematics and computing, Psychology, Psychology

## Abstract

The growth of food and alcohol marketing on social media creates a need for scalable monitoring methods that go beyond manual processing. This study evaluates whether Large Language Models and Vision-Language Models can recognize advertisements and identify their features in consistence with general public or expert opinion. We collected 1000 Facebook ads from major Belgian brands, and annotated them with 600 crowd workers, three dieticians and four AI models (GPT-4o, Qwen 2.5, Pixtral and Gemma3). Our analysis of the data shows that for single-option advertisement features, like alcohol presence or target group, GPT-4o and Qwen reached agreement with the dietician consensus above 90%, similar to the level of pairwise agreement observed between individual dieticians. Though agreement was lower for multiple choice features, like premium offers and marketing strategies, it was still within the variability observed in crowd raters. The bias analysis revealed how models interpret certain labels, with some being consistently under- or over-detected. Based on these findings, we propose tiered deployment recommendations that distinguish between ad features that MLLMs can already monitor with human-level accuracy, and more complex features requiring expert oversight and taxonomy refinement, like marketing strategies or food categories.

## Introduction

Contemporary marketing practices, including advertising on social media, and the limited regulation of such advertising, has created a need for reliable and scalable methods to monitor and control the exposure. Understanding and regulating unhealthy food and alcohol advertising, in particular targeted to children, adolescents, and young adults, has long been a public health priority, particularly due to its influence on consumer behavior and dietary patterns^1^.

Traditionally, exposure monitoring has been relied on recognition and identification of advertisements (further *ads*) by human annotators. Particularly, expert dieticians annotate and label advertising images and captions to alert consumers about the healthy or harmful nature of food products and beverages. To allow in future automated recognition and labelling of products, the existent annotations have been used to train supervised machine learning (ML) or deep learning (DL) models.

More recently, the emergence of large language models (LLMs) has transformed many natural language processing (NLP) routines, for instance, text classification, with promising results^2^. This has led to growing interest in validating LLM performance against human judgment, either through crowd-sourced annotators or domain experts, such as dieticians, to assess their reliability and consistency. Building on this, multimodal and vision-language models (VLMs) have been introduced to process both images and text, offering new opportunities for automated ad classification, and hence larger scale monitoring of exposure to advertising. The knowledge gap that the present paper aims to close is to check whether the AI systems agree with general public and/or expert dietician opinions when labeling advertisements with multiple complex properties.

In the past decade, the application of Artificial Intelligence (AI) has considerably increased for analyzing and categorizing food and beverage ads. The first approaches have largely focused on developing supervised ML models to automate the identification of ad characteristics, typically requiring labeled training data, manually annotated by human experts. For example, ML models were used to detect persuasive marketing techniques in food advertisements across digital platforms, showing that automated tools can assist in monitoring unhealthy food promotion^3^. Similarly, a DL pipeline was proposed to classify fast-food ads based on visual appeal and emotional framing^4^. Another study developed a convolutional neural network model to detect sugar-sweetened beverage ads in social media feeds^5^.

Other work extended these techniques to outdoor advertising, where image data from billboards and urban environments were used as input. For instance, object detection and image classification methods were used to identify branded food and alcohol ads in public spaces and near schools^6^. These approaches, while useful, were limited by their dependence on large, high-quality labeled datasets and their inability to generalize beyond the scope of training data.

With the current emergence of LLMs and Generative AI, there has been a rapid shift in how researchers and practitioners approach ad classification and content analysis. The advantage of the LLMs is that the models can produce product labelling without or with just a few hinting examples for the labels in the classification requests, further referred to as *prompts*. This has opened new possibilities for scalable and flexible annotation, especially in domains where manual labelling is resource-intensive and time-consuming.

Human annotations - whether from domain experts or crowdsourced workers - remain the reference standard for evaluating automated classification. In advertising research, expert coders (e.g., dieticians in nutrition research or food policy studies) provide subtle but important interpretations that general-purpose models may miss, while crowdworkers enable large-scale labelling at lower cost. Recent work has increasingly evaluated the validity of LLM-based classifications by comparing them to human judgments. For instance, close alignment was found between LLMs and expert coding of ideological frames in political texts^7^. Other studies similarly demonstrated that LLMs can match or even exceed crowd workers in accuracy and consistency for a variety of text analysis tasks^8,9^. However, these findings often depend on task complexity, prompt design, and the specificity of the label set. It’s also important to highlight that sometimes, while LLMs perform well in general classifications, they may struggle with domain-specific expertise, reinforcing the importance of benchmarking against expert-coded data^10^.

A smaller number of studies directly compared AI outputs to both expert and crowd benchmarks within the same task. For example, with careful configuration, open-source LLMs approached trained annotator performance and often surpassed crowd accuracy in a dual-benchmarking setting^11^. Yet such dual-benchmarking studies remain rare, particularly for multi-label and policy-relevant classification tasks such as food and alcohol advertising, where a single ad can have multiple attributes (e.g., product type, marketing strategy, health category).

Building on the success of text-based LLMs, a new generation of models has emerged that integrate visual and textual modalities, commonly referred to as vision–language models (VLMs) or multimodal large language models (MLLMs). These models are capable of processing both images and text inputs, and have demonstrated strong performance in tasks such as misinformation detection, hate speech detection, and social context generation^12,13^. In domains such as stance detection, VLMs have achieved high agreement with human annotators, approaching the performance of fine-tuned supervised models^14,15^. However, even these efforts typically benchmark against either expert or crowd annotations in isolation, not jointly across both.

To our knowledge, this form of dual benchmarking has not yet been systematically applied to VLMs or MLLMs in the context of structured, policy-relevant ad annotation. While some studies have reported promising agreement rates between VLM-generated and human labels, these comparisons typically rely on either expert or crowd benchmarks in isolation^16^. As a result, little is known about how AI models perform across different human reference standards, or how conclusions about reliability and bias depend on the benchmark choice, particularly in policy-relevant contexts like food, non-alcoholic beverage, and alcohol advertising. Methodologically, this study introduces a dual-benchmarking design by directly validating multiple MLLMs against both crowd-sourced annotators and domain experts within the same annotation framework. This design enables systematic, head-to-head comparison across all rater types, while also presenting benchmark-specific analyses like bias diagnostics and simulation-based contextualisation. Beyond benchmarking, we synthesize these results into actionable guidance for AI-assisted ad monitoring, clarifying where current models can be reliably deployed, where human oversight remains necessary, and how bias evaluation should inform policy-relevant applications. This leads us to the following research questions:

1. **Expert Agreement**: To what extent do various MLLMs align with human crowd and domain experts (dietician) consensus when coding multiple features of food and alcohol advertisements?
2. **Question Complexity**: How does agreement differ between single-option and multi-option question formats?
3. **AI Bias**: What systematic patterns of (dis)agreement emerge across models and question types, and do certain models or label categories show consistent biases or strengths?

## Results

### Pairwise agreement

**Fig. 1:**
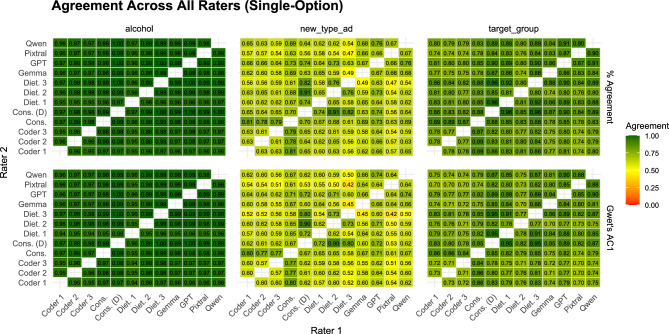
Pairwise agreement between crowd coders, dieticians, consensus labels, and AI models for single-option variables. Values represent both proportion agreement (top) and Gwet’s AC1 (bottom). For comparisons involving dieticians, agreement is computed over 400 ads; all other comparisons are based on the full set of 1000 ads.

Figure 1 presents the agreement heatmap for the three single-choice variables (*Alcohol*, *Ad Type*, and *Target Group*).

Agreement levels varied considerably by variable. For the *Alcohol* variable, agreement was nearly perfect across all coders, with both percentage agreement and Gwet’s AC1 consistently above 0.95, reflecting the low ambiguity of this binary task. Agreement was lower for *Target Group*, typically between 0.75 and 0.9, and the lowest agreement was observed for *Ad Type*, where values ranged between 0.5 and 0.7, even among dieticians. Importantly, AI models performed comparably to human coders across all three variables, suggesting that observed disagreements largely reflect task complexity rather than systematic model underperformance.

**Fig. 2:**
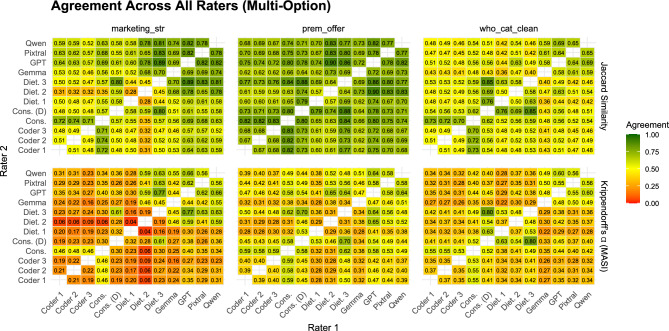
Pairwise agreement between crowd coders, dieticians, consensus labels, and AI models for multi-option variables. Values represent Jaccard similarity (top) and Krippendorff’s Alpha with MASI distance (bottom). For comparisons involving dieticians, agreement is computed over 400 ads; all other comparisons are based on the full set of 1000 ads.

Agreement was notably lower for the multi-label tasks than for the single-choice variables, reflecting the greater complexity of these classifications. As seen in Figure 2, for *Premium Offers*, agreement was relatively high, with Jaccard values in the $0.65-0.80$ range and corresponding Krippendorff’s $\alpha$ around $0.4-0.6$, indicating moderate but consistent alignment across coders. In contrast, *Marketing Strategies* and *WHO Food Categories* showed weaker agreement: Jaccard similarities typically ranged between 0.4 and 0.6, while $\alpha$ values were often below 0.4, even among dieticians.

When comparing human coders, agreement between crowd consensus and dieticians was generally strong, though not uniformly higher than AI-dietician alignment. Inter-dietician agreement was highest on the single-choice variables, often above 0.80, while on multi-label questions it dropped to around $0.35-0.45$. Interestingly, the lowest agreement across human coders (both dieticians and crowd) was observed in *Marketing Strategies*, sometimes going as low as 0.04 (dietician 1 - dietician 2).

**Fig. 3:**
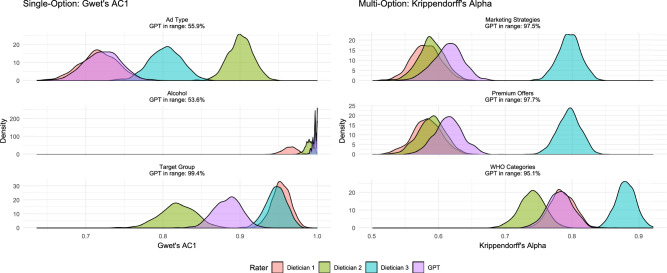
Bootstrap validation of AI-human agreement across coding tasks. Distributions show 1000 bootstrap resamples of Gwet’s AC1 (single-choice questions, left) and Krippendorff’s Alpha (multi-label questions, right) for three expert dieticians and GPT-4o relative to consensus labels. The “GPT in range” percentages indicate the proportion of bootstrap samples where GPT-4o’s agreement falls within the min-max range of the three human experts. Higher overlap between GPT-4o and dietician distributions indicates comparable performance and provides evidence of exchangeability.

The Monte-Carlo simulation results shown in Figure 3 provide further evidence for the reliability of GPT’s ad annotation performance. Across all multi-option questions, GPT’s agreement coefficients fell within the range of dietician variability in $95.1\%$ to $97.7\%$ of bootstrap simulations. For single-option questions, although lower, GPT’s still agreement still fell within the range of human reliability at least $53.6\%$ of simulations. This indicates that GPT’s performance is statistically indistinguishable from human performance. Bootstrap-based uncertainty estimates for GPT’s agreement coefficients, including median values, $95\%$ bootstrap confidence intervals, and the proportion of simulations in which GPT falls within the dietician agreement range, are reported in Table S6 in the Supplementary Material.

### AI bias analysis

**Fig. 4:**
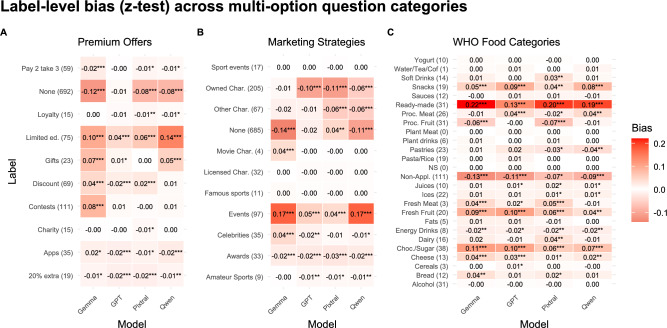
Label-level bias in AI model predictions for multi-option questions compared to human consensus. Panels show the difference in selection rates between each AI model and human consensus for **Panel A:** *Premium Offers*, **Panel B:** *Marketing Strategies* and **Panel C:** *WHO Food Categories*. The cells show option bias (mean selection rate difference: model - human) with z-test significance (*$p < 0.05$, **$p < 0.01$, ***$p < 0.001$), where crowdsourcers consensus was used for *Premium Offers* and *Marketing Strategies* and dietician consensus was used for *WHO Categories*. Positive values indicate over-selection; negative values indicate under-selection. The numbers in the brackets next to each label represent the number of ads in which the label was selected by the reference consensus out of the total sample of 1000 ads for crowd workers and 400 ads for dieticians. Note that the category *Other* for *Premium Offers* (**Panel A**) and *Marketing Strategies* (**Panel B**) was only available as an option to human coders and dieticians, not to AI models, so it was not included in the bias analysis.

Figure 4 shows the bias analysis results for the multi-option questions: *Premium Offers*, *Marketing Strategies*, and *WHO Food Categories*. Heatmaps for the single-option variables are provided in Supplementary Fig. S3.

For *Premium Offers*, the most pronounced pattern was the consistent under-detection of *None* and over-detection of *Limited Edition* offers across all four models. The z-test showed that Qwen over-detected *Limited Edition* by $14\%$ compared to crowd consensus, while Gemma demonstrated the strongest under-detection of *None* at $-12\%$. Several categories show more modest and inconsistent biases across models, with *Loyalty*, *Charity* and *Apps* displaying minimal bias for most models, suggesting these promotional types are more reliably identified across different AIs.

For *Marketing Strategies*, *Events* was the most problematic category, with all models demonstrating substantial over-detection, reaching $17\%$ for both Gemma and Qwen. Similar to the *Premium Offers* pattern, *None* is again significantly under-detected, especially by Gemma ($-14\%$) and Qwen ($-11\%$). Character-based marketing strategies present another challenge, with *Owned Characters* and *Other Characters* being systematically under-detected by three of the four models by $6-11\%$.

The *WHO Food Categories* analysis shows the most extreme biases found in all classification tasks, with *Ready-made* meals showing significant over-detection ranging from $13\%$ (GPT) to $22\%$ (Gemma). *Non-applicable* ads follow the previous pattern of under-detection across all models, with biases ranging from $-7\%$ (Pixtral) to $-13\%$ (Gemma). Multiple food categories exhibit notable over-detection, including *Fresh Fruit* ($4-10\%$), *Chocolate/Sugar* products ($6-11\%$), and *Snacks* ($4-9\%$).

Across the three multi-option questions, several consistent patterns emerge that highlight systematic AI biases. Most notably, the persistent under-detection of “None” or “Non-applicable” categories across all models suggests a general tendency for AI systems to over-classify rather than acknowledge the absence of relevant content (generate false positives). Additionally, Gemma and Qwen consistently demonstrate the most extreme biases, while GPT shows more moderate and conservative classification patterns. The magnitude of biases appears to increase with classification complexity, with WHO Food Categories showing the largest individual coefficients, suggesting that more granular categorization tasks amplify AI classification errors.

The smaller analysis of 100 outdoor ads annotated by all four AI models and the three dieticians (presented in Section 5 in Supplementary Material) supported these findings, revealing similar model-dietician alignment across ad delivery formats (online vs. outdoor). The discussion that follows places these results in the context of the larger body of literature and examines their implications for AI-based ad monitoring.

## Discussion

This study evaluated whether LLMs and VLMs can reliably classify food and alcohol ads when benchmarked against both expert (dietician) and crowd-sourced human coders. Across single-option and multi-option tasks, AI-human agreement often approached the level of human-human agreement. Importantly, variability among dieticians themselves was sometimes as large as, or even larger than, the differences between AI models and human coders, suggesting that disagreements are not unique to AI but are inherent to the complexity of the task.

**Expert-Crowd-AI Agreement**. Our results show that large language models can come very close to human-level performance on single-option tasks (Figure 1). For example, on the *Alcohol* variable, agreement between all AI models and the dieticians was virtually indistinguishable from the agreement observed among the dieticians themselves. Similarly, for *Target Group*, which is a more subjective question, models still achieved agreement comparable to or only slightly below human-human agreement. These findings suggest that for relatively unambiguous dimensions, LLMs reach a standard comparable to trained human coders (**RQ1**). Importantly, the variability between dieticians themselves often matched or exceeded the differences between models and dieticians, indicating that some level of disagreement is inherent even among experts.

Agreement was consistently lower on multi-option variables (Figure 2) compared to single-option ones, reflecting the added complexity of coding features that can co-occur (**RQ2**). At the same time, agreement coefficients must be interpreted with caution, as all the multi-label questions involved a large number of possible response categories (see Table 1). As noted before, the number and prevalence of categories directly influence attainable $\kappa$ and $\alpha$ values, making standard thresholds inappropriate^17^. This is why in our case, $\alpha$ values of 0.45 in *WHO Categories* (GPT - Diet. Consensus) and of 0.65 in *Premium Offers* (GPT - Dietician 2) are substantially above random-like agreement, and consistent with the empirically estimated upper bounds for this task type (Supplementary Section S5).

**Table 1:** Description of the analysed questions.

Question	Single/Multi	# choices	Examples
Alcohol	Single-option	2	Yes/No
Target Group	Single-option	3	Child/Adolescent/Adult
Ad Type	Single-option	9	Manufacturer, Restaurant, Retailer, etc.
Premium Offers	Multi-option	10 (+*Other*)	Price discount, Limited edition, Loyalty programs
Marketing Strategies	Multi-option	11 (+*Other*)	Promotion of sport/cultural events, Use of cartoons/celebrities
WHO Food Categories	Multi-option	26	Dairy, Processed Fruit, Snacks, Soft Drinks

A particularly striking result was the very low inter-dietician agreement on marketing strategies. This pattern likely reflects the professional background of the expert coders: dieticians are specifically trained to identify and classify nutritional content, but not to consistently detect abstract or context-dependent marketing strategies. Unsurprisingly, dietician agreement was higher for *WHO Categories* than for *Marketing Strategies*, and nearly similar for *Premium Offers* (when accounting for chance), where the difference between non-nutritional features such as for example *Gifts* and *Events* was more difficult to grasp. These findings emphasize that even expert benchmarks vary in reliability depending on the domain of expertise, and sometimes an average human can classify these dimensions at least as well as AI models or trained experts.

It is also important to distinguish between raw agreement measures (proportion agreement and Jaccard similarity) and chance-corrected coefficients (Gwet’s AC1 and Krippendorff’s alpha). Raw agreement provides an intuitive picture of overlap and is particularly informative for single-option variables with balanced distributions, but it can overstate reliability when some categories dominate. In multi-option contexts, where rare categories increase the likelihood of incidental overlap, chance-corrected metrics account for probability of agreement by chance. In our results, Jaccard similarities for multi-label tasks were often moderate, for example GPT’s highest agreement with human coders was 0.89 in *Marketing Strategies*, 0.9 in *Premium Offers* and 0.63 in *WHO Categories*, while corresponding $\alpha$ values were lower ($\approx 0.48-0.77$), reflecting the stricter criteria imposed by accounting for chance. These patterns highlight the fact that lower chance-corrected values do not necessarily signal poor reliability, but rather the higher difficulty of the coding task. For this reason, scholars should select agreement coefficients carefully, report both raw and chance-corrected measures, and interpret them relative to the complexity of the task and the distribution and number of categories.

**AI Bias Analysis.** In addition to differences in agreement magnitude, the analysis of disagreement patterns offers further insight, which is captured by the AI bias analysis (**RQ3**). For nutrition-related questions, we use the dietician consensus as the reference benchmark, reflecting their domain expertise. For *Marketing Strategies* and *Premium Offers*, however, inter-dietician agreement was substantially lower than agreement among crowd coders (Figure 2). Rather than indicating a failure of expert judgement, this pattern reflects a domain mismatch, since the dieticians were recruited as nutrition experts, not as marketing or advertising specialists, and these constructs lack a single, well-established professional taxonomy. For these marketing-oriented variables, we therefore do not treat any benchmark as a “*gold standard*”. Instead, the crowd consensus is used as a behaviourally grounded reference that captures how promotional cues are interpreted by non-expert consumers, who represent the population to whom such advertising is primarily targeted. Importantly, the low inter-dietician agreement itself constitutes a substantive finding, highlighting the inherent ambiguity and subjectivity of these tasks and placing an upper bound on attainable agreement for both human and AI annotators.

At the label level, the bias analysis reveals systematic, model-specific patterns. For example, Qwen showed pronounced under-detection of certain WHO food categories such as pastries and sauces, while Gemma more frequently missed several processed food categories, including processed meats and processed fruits and vegetables. Pixtral performed comparatively better on some categories, such as dairy products, whereas GPT, despite stronger overall performance, showed weaker detection of discounted offers and endorsements involving celebrities or charities. Such category-specific weaknesses mean that two models with similar global agreement scores may nevertheless diverge in the types of errors they make. From a monitoring perspective, this raises concerns that exposure to specific product categories or marketing techniques may be systematically under- or overestimated if only a single AI model is relied upon.

More concretely, these biases have direct implications for real-world monitoring and policy applications. For example, the systematic under-detection of the implicit *None* category for Premium Offers implies that the prevalence of promotional marketing would be overstated in large-scale analyses, potentially exaggerating the intensity of persuasive advertising practices. Similarly, the strong over-detection of *Events* within Marketing Strategies may lead to inflated estimates of campaign sophistication, while the concurrent under-detection of character-based strategies risks obscuring precisely those techniques that are most often subject to regulatory scrutiny. This is particularly relevant in policy contexts related to copyright and intellectual property, where the unauthorized or restricted use of licensed or owned characters is explicitly regulated and systematic under-detection could lead to false conclusions about compliance. In the context of WHO Food Categories, the pronounced over-detection of ready-made meals and other ultra-processed food categories yields conservative estimates of unhealthy food exposure, which may be acceptable for screening purposes but problematic for accurate prevalence estimation or policy evaluation.

Although there were minor differences in agreement patterns between Dutch and French ads across different rater pairs and question categories, these differences were not statistically significant and insufficient to draw meaningful conclusions about language-specific variations. The predominantly positive delta values (indicating higher agreement for Dutch content) suggest possible language effects, but the lack of statistical significance indicates that AI models perform similarly across both languages in this ad classification context. The full results can be found in Supplementary Fig. S4.

Taken together, these results highlight both the feasibility and the limitations of using AI in food and alcohol ad monitoring. Across models, GPT and Qwen showed the strongest and most consistent alignment with dietician consensus, often matching the range of inter-dietician agreement on single-option variables and approaching it on the more complex multi-option tasks. Pixtral showed moderate but uneven performance, while Gemma was the weakest overall, though occasionally stronger on specific categories.

**Actionable Guidelines.** Based on the agreement analyses, bias exploration and the simulation-based contextualisation of multi-option agreement, we propose a set of actionable guidelines for deploying AI in food and alcohol advertising monitoring. Rather than treating AI performance as uniform across tasks, our results support a tiered strategy that differentiates between tasks that are already suitable for automation, tasks that require cautious, context-dependent use, and tasks that remain unsuitable for deployment or require future work. These guidelines are intended to support researchers and public health monitoring agencies in making task-specific, evidence-based decisions about when and how AI models can be responsibly deployed in advertisement annotation.

1. AI models can already be deployed with relatively high confidence under similar single-label task conditions. In particular, *Alcohol* identification showed near-perfect agreement across models and benchmarks, minimal bias and consistency across languages. We also place *Ad Type* and *Target Group* within this tier, as the agreement was consistently high and within the range of inter-dietician reliability. Although some minimal tendencies were detected within the *Ad Type* question, these three single-option tasks can be reliably automated for public health monitoring, offering substantial reductions in manual coding effort. Before operational deployment, we recommend conducting a small-scale local validation study comparing AI outputs with at least two independent human annotators using the same taxonomy and prompt structure, as agreement levels are task- and label-set dependent.
2. All multi-option questions in this study fall into a second tier that requires more careful deployment and oversight. Although AI models frequently achieved agreement levels comparable to individual human coders and often within their range, these tasks exhibited greater sensitivity to label ambiguity, benchmark choice and systematic bias. For these variables, AI should be used as an as assistive annotation tool, for example to pre-screen ads, prioritize human review, or support sensitivity analyses. Importantly, part of the observed disagreement reflects the ambiguity and sparsity in the label definitions rather model performance alone, as shown by inconsistent human coding. For these tasks, reconfiguration of the label sets (e.g., collapsing rare or abstract categories) may be required to enable reliable automation. Researchers are further advised to evaluated biases on a case-by-case basis, select benchmarks aligned with domain expertise (e.g., dieticians for nutrition-related questions, marketing researchers for marketing constructs, etc.), and interpret agreement magnitudes using contextual references. The simulation study presented in Section 5 of Supplementary Material provides guidance for interpreting agreement in high-dimensional, sparse multi-option tasks, where conventional threshold may not be appropriate.
3. Several potentially relevant targets remain unsuitable for reliable AI deployment at present. These include domains not evaluated in this study, such as tobacco or other harmful substances, as well as fine-grained nutritional attributes (e.g., macro-nutrient composition). Given the absence of validation evidence and the increased semantic complexity of these variables, automated outputs in these area should currently be treated as exploratory.

**AI Model Selection.** In addition to methodological considerations, selecting an AI model involves significant trade-offs. Cost, availability, model size and computational efficiency are some of the variables that must be considered when evaluating performance. Commercial APIs can classify ads quickly and accurately, but they may also involve higher financial costs and potential privacy concerns. GPT-4o and Pixtral were the two AI models that we used through the API, and while both showed similar fast processing times, GPT performed considerably better. Although they frequently demand more computational power and longer processing times, open-source models provide more transparency and can be used in closed environments to protect sensitive data. So the other two models, Gemma3 and Qwen2.5, while slower in ad classification, still showed decent accuracy and were deployed locally with no costs involved. These trade-offs imply that there is no single “best” model that works in all situations. For example, smaller or API-based models may be better in large-scale public health monitoring, where cost and efficiency are critical, while locally deployed open-source models may be preferable in research or policy settings, where privacy requirements are stricter.

In summary, this study shows that the performance of LLMs and VLMs in food and alcohol ad annotation is strongly task-dependent. AI models reach human-level performance on well-defined single-option tasks, indicating that such dimensions can already be reliably automated in large-scale ad monitoring.For more complex multi-option tasks, agreement is substantially lower and bounded by label ambiguity, benchmark choice, and the structure of the annotation taxonomy. The combined use of agreement metrics, label-level bias analysis, and simulation-based contextualisation highlights that lower agreement coefficients in these settings reflect task complexity rather than model failure alone. Taken together, these findings clarify both the feasibility and the limits of AI-assisted ad monitoring, and motivate the tiered, bias-aware deployment guidelines proposed above.

Several limitations should be noted. First, all AI models were prompted exclusively in English, while ads appeared in Dutch, French, or English. Although this guarantees consistent task framing, it introduces a cross-lingual processing component that may affect model performance, especially for smaller open-weight models. Second, several annotation tasks (marketing constructs) reflect existing survey procedures rather than a fully standardized taxonomy, and the low agreement observed for these variables emphasizes the need for more conceptual refinement. Finally, this study focused on a set of Belgian food and alcohol ads, and future work should extend validation efforts to additional substances, locations, and social media platforms.

## Methods

**Fig. 5:**
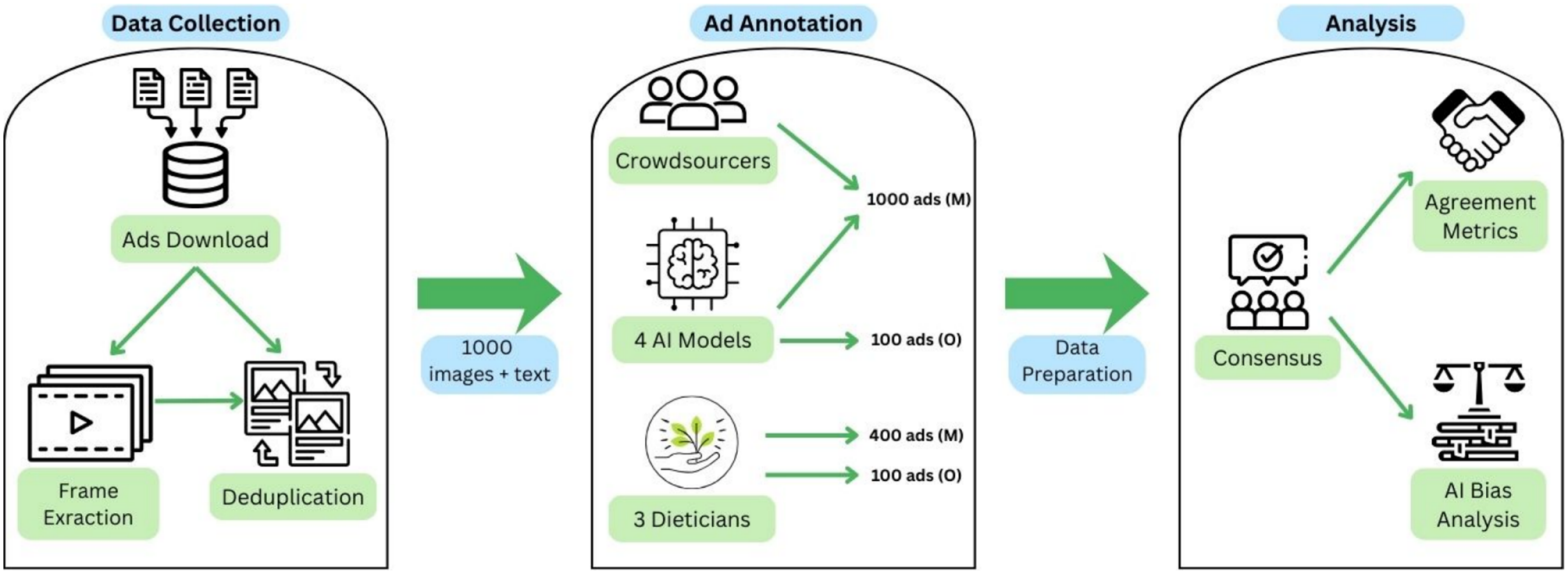
Overview of the Research Design. The Methodology is divided into three key stages: Data Collection, Ad Annotation, and Analysis. The process began with the download of ad data, followed by image extraction from videos and deduplication, and selection of 1000 unique Meta (M) ad images and associated text. Annotations were collected from approximately 600 crowd workers and four AI models (GPT-4o, Qwen 2.5, Pixtral, and Gemma3) across all 1000 (M) ads, and independently by three expert dieticians on a 400 (M) ad subsample. Additionally, analysis was conducted on 100 outdoor (O) ads, which were classified by the three dieticians and all four AI models. The Analysis stage involved deriving consensus labels (as reference) for both crowd and expert annotations, which were then used to evaluate the AI models using agreement metrics (Gwet’s AC1 and Krippendorff’s Alpha) and systematic bias analysis.

The methodology for validating the AI models is summarized visually in Figure 5. The study used a dual-benchmarking approach, comparing four MLLMs against both a sample of general audience crowd-sourcing workers and a smaller group of expert dieticians. The overall design consisted of three stages: (1) Data Collection, which involved retrieving, processing and deduplicating 1000 social media ads (consisting of images + captions); (2) Ad annotation, where all 1000 ads were labelled by crowd workers and the four AI models, and a 400 ad subsample and 100 outdoor ads were labelled by dieticians and the four AI models; and (3) Analysis, which included data preparation, derivation of consesnsus labels, and included calculation of human-AI agreement and bias analyses.

### Data collection

We began by selecting 132 major Belgium brands based on Euromonitor market share data, covering various food areas, such as: fast-food restaurants, alcohol brands, supermarkets, food delivery companies, and brands of specific manufacturers. Ad data, including both metadata and media, was retrieved from the Meta Ad Library^18^ using the AdDownloader package^19,20^ for the period between July 2023 and July 2024. To create a varied sample and unique sample of 1000 ads, videos were converted to images by extracting a frame every 2 seconds, and exact duplicates were removed by computing an MD5 hash over the image pixel data, a standard content-based approach for exact data deduplication^21^. Finally, we selected a random sample of 1000 deduplicated ad images and metadata, covering 77 brands. An example of a Meta ad is shown in Supplementary Fig. S1. The outdoor ads were retrieved from an earlier study on outdoor advertising around schools in Flanders where images of ads were taken by fieldworkers^22^.

### Human labelling procedure

To establish reliable human benchmarks for comparison with AI classifications, we collected annotations from two different groups of human coders: approximately 600 crowd-sourced participants each coding five (M) ads, and three expert dieticians each coding 400 (M) ads and 100 (O) ads. Both groups completed the same core classification task, following the same instructions (which can be found here), definitions, and coding guidelines.

We confirm that all methods were carried out in accordance with all relevant guidelines. The research protocol was reviewed and approved by the Ethical Review Committee Inner City Faculties (ERCIC) at Maastricht University. Informed consent was obtained from all participants prior to data collection, including both crowd workers recruited through online platforms and the expert dieticians. Participation was voluntary and anonymous, and no personally identifiable data were collected.

#### Ad classification task

The ad classification task, hosted on Qualtrics, required coders to evaluate the ad image, accompanying caption and the brand. They answered a series of structured questions designed to capture six key ad features, which fall into two categories:

1. Single-Option Variables (select **only one**): *Alcohol* presence (Yes/No), *Target Group* (Adult/Adolescent/Child), and *Ad Type* (Manufacturer, Restaurant, etc., 9 options in total).
2. Multi-Option Variables (select **multiple**): *Premium Offers* (Discounts, Contests, etc., 10 options in total), *Marketing Strategies* (use of celebrities, sport events, etc., 11 options in total), and *WHO Food Categories* (dairy, processed meat, etc. 18 options in total).

An example of how the questions were formulated can be found in Supplementary Fig. S2.

Participants were provided with a detailed instructions document that outlined definitions and examples for each question of the classification task. The *WHO Food Categories* required a subsequent classification of the level of food processing (unprocessed, ultra-processed, etc., 5 options in total) for each selected category.

#### Crowdsourcing participants

Crowd coders were recruited from the general adult populations of the Netherlands, Belgium, and France. Inclusion criteria required participants to be at least 18 years old, reside in one of these countries, and be proficient in Dutch, French, or English. Recruitment was conducted via Prolific, a widely used online crowdsourcing platform. After providing informed consent, participants were directed to a Qualtrics-hosted survey. The survey collected demographic information, followed by the Ad Classification Task, and finally, additional questions regarding nutrition knowledge and social media usage. In-built counters in the survey interface tracked the number of annotations per ad, as well as the IDs of ads that a crowd worker is shown, and made sure each ad received annotations from 3 distinct crowd workers, and no worker saw the same ad twice.

To maintain data quality, three randomized attention-check questions were included, which were designed to identify inattentive respondents and maintain the integrity of the classification dataset^23^. Participants who failed more than one of these checks were rejected.

#### Nutrition domain “experts”

Three registered dieticians with formal training in nutrition were recruited to provide a domain-specific benchmark for nutrition-related variables. All three dieticians were recent graduates in nutrition and were therefore considered domain experts for food and health classifications, but not for marketing-related variables. They annotated the 400 (M) subsample and all 100 (O) ads. The dieticians followed the same procedural guidelines and Ad Classification Task as the crowd-workers, with the exception of the attention checks. Each of the dieticians worked independently and filled in the survey using the language of their choice (French/Dutch), and all ads were evaluated in their original language. Finally, the dieticians had no competing interests and no ties to any of the brands included in the annotations.

### AI classification

In addition to human annotations, we obtained classifications from four MLLMs capable of processing both textual and visual inputs. This allowed us to evaluate model performance in a setting that closely mirrors the human task, where both the ad image and its caption are present.

#### AI models

All models were used in their base form without fine-tuning, task-specific adaptation, or activation of reasoning modes. Instead, they were directly prompted to perform the classifications using the same instructions provided to human coders. Two models were accessed via their APIs, while two open-weight models were run locally using the Transformers Python library^24^. Both open-weight models were run locally on Maastricht University’s Data Science Research Infrastructure (DSRI) using NVIDIA GPUs.

1. **GPT-4o-2024-08-06** (OpenAI) - A closed-source multimodal model accessed via the OpenAI API. It was selected for its strong general-purpose performance and ability to process ad images alongside captions and brand names. (https://openai.com/index/hello-gpt-4o/)
2. **Pixtral-12B-2409** (Mistral AI) - A free-access VLM made accessed via the Mistral API. While smaller in scale than GPT-4o, it was included to assess how open-access, lower-cost models perform on the same classification task. (https://mistral.ai/news/pixtral-12b)
3. **Qwen2.5-VL-32B-Instruct** (Alibaba Cloud) - A high-capacity open-weight multimodal model. Its large size and open-weight availability make it a relevant benchmark for reproducible academic research. (https://huggingface.co/Qwen/Qwen2.5-32B-Instruct)
4. **Gemma-3-12B-it** (Google DeepMind) - Another open-weight model from a newer generation of Google’s Gemma family. While smaller than Qwen2.5, it is designed for efficient inference without sacrificing multimodal capabilities. (https://huggingface.co/google/gemma-3-12b-it)

For all models, inference was performed using deterministic decoding (temperature=0) without sampling (do_sample=False). Prompt lengths were kept within each model’s context window, and no dynamic truncation strategies were applied. By including both proprietary and open-weight models and applying identical instructions across all systems, performance differences reflect out-of-the-box model behavior rather than differences in training, adaptation, or evaluation setup. Additional implementation details are provided in Section 1 of the Supplementary Material.

#### AI Ad classification task

The AI classification was implemented in Python. The models were provided with detailed task instructions and label definitions, outlined in Section 1.1 of the Supplementary Material. The classification relied on instruction-based prompting with detailed label definitions, consistent with standard zero-shot usage of large language models^25^. Although illustrative descriptions (e.g., “a Coca-Cola bottle in someone’s hand”) were included to clarify label semantics, these serve as definitional guidance rather than labelled examples and therefore do not constitute “shots”.

The prompting strategy was designed to simulate the guidelines provided to human coders, while presenting each feature as an independent binary decision, a common strategy in multi-label learning that improves interpretability and tractability at the cost of potential error accumulation across labels^26^. The models were instructed to respond using a strict standardized format (*QUESTION_LABEL*: Yes/No - Brief explanation), to ensure consistent and easily readable outputs. Instead of using external structured-output frameworks, this format was enforced through prompt system instructions and validated through deterministic post-processing. The brief explanations accompanying each Yes/No answer were intended to encourage grounded responses and to simplify manual inspection during prompt pre-optimization, consistent with prior work on explanation-based prompting^27^; they were not used in the quantitative analysis.

Through manual inspection of model outputs, the prompt was iteratively refined on a small sample of ads, prior to starting the full classification pipeline. This pre-optimization step focused on clarifying instructions, addressing ambiguities in label definitions, and ensuring stable adherence to the required output format. During the main analysis, a single fixed prompt was applied consistently to all models and ads, without the use of systematic prompt engineering or model-specific tuning.

**Fig. 6:**
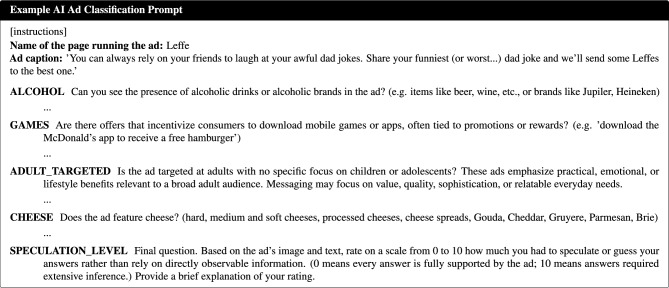
Example of the AI ad classification prompt. The ad caption was kept in its original language, which can be either English, Dutch or French. The prompt instructions and sub-questions were kept only in English. The figure shows the structured instruction format used for all models, where each label category (e.g., *Games* and *Limited Edition* for *Premium Offers*, or *Cheese* and *Snacks* for *WHO Categories*) was presented as a separate question requiring a Yes/No answer with a brief justification. Standardized definitions ensured consistency and comparability with human coders.

All prompts were provided in English, irrespective of the original language of the ad text, while the models received the original ad images and captions without translation. This design choice ensured consistent task framing across models but introduces a cross-lingual processing component, which is considered when interpreting model performance differences. Figure 6 presents an example of the prompt structure.

### Data preparation

The analysis focused on six key variables relevant to food and alcohol marketing. Three were single-option questions: (1) Ad Type, (2) Target Group, and (3) Is Alcohol. The other three were multi-option variables: (4) Premium Offers, (5) Marketing Strategies, and (6) WHO Food Category. A summary of the questions is presented in Table 1.

To prepare the data, we performed label harmonization and quality checks. For alcohol ads, additional preprocessing was applied to adjust inconsistent annotations in the Ad Type and WHO Category variables - for example, reclassifying food categories or types when alcohol was a featured product.

**Consensus** labels were then derived for each question type. For single-option variables, we used a majority vote across the three human coders. In the event of a tie, the first occurring label was selected. For multi-option variables, we applied a frequency threshold approach: all labels chosen by at least two of the three coders were included in the consensus. If no label met this threshold, we used the union of all labels selected by any coder (*threshold-plus-union*), ensuring that at least some agreed-upon content was retained. A sensitivity analysis comparing alternative consensus rules (union-only, intersection-only, threshold-only) was conducted and is reported in Section 7 of the Supplementary Material. The results show that the main agreement patterns are generally robust to reasonable variations in the consensus rule, with minimal differences in agreement when no fallback is applied to the threshold-only rule. These labels served as the reference standard for evaluating AI-human agreement: crowd-worker consensus for the full sample of 1000 ads, and dietician consensus for the subsample of 400 ads.

### Pairwise agreement

The main analysis focused on pairwise agreement between crowd coders, dieticians and AI models for each of the six questions. Various agreement metrics were computed based on the type of the question. All data analysis was done in R. For variables where each coder (human or AI) selected exactly one category, we used three metrics:

1. *Proportion Agreement* (raw agreement rate) - the simplest measure, indicating the proportion of cases in which two coders gave exactly the same label^28^:
  1 $$\begin{aligned} P_o = \frac{\text {Number of items where coders agree}}{\text {Total number of items}} \end{aligned}$$
  Although easy to interpret, it does not account for the agreement expected by chance.
2. *Gwet’s AC1* - a chance-corrected coefficient (^28–30^), that uses the same general chance-adjusted formula, but calculates the expected agreement differently, given by:
  2 $$\begin{aligned} p_e = \frac{1}{k} \sum _{i=1}^k p_i (1 - p_i) + \sum _{i=1}^k p_i^2 \end{aligned}$$
  Unlike Kappa^31^, AC1 is less sensitive to class prevalence and imbalance, which can lead to the Kappa paradox, where high observed agreement yields low Kappa values^32^. In datasets with skewed label distribution, which is common in our ad classification task, AC1 produces more stable and representative estimates of agreement.

For variables allowing multiple categories per item, we used both set-based similarity measures and chance-corrected agreement statistics:

1. *Jaccard Similarity*- measures the intersection over the union of two coders’ label sets, given by the formula^33^:
  3 $$\begin{aligned} J(A, B) = \frac{|A \cap B|}{|A \cup B|} \end{aligned}$$
  This metric provides an intuitive and easy to interpret value between 0 (no shared labels) and 1 (identical label sets), but it does not correct for chance agreement.
2. *Krippendorff’s Alpha using MASI Distance* - Krippendorff’s Alpha^34^ is a chance-corrected reliability coefficient that can handle various data types and missing values. It is computed as:
  4 $$\begin{aligned} \alpha = 1 - \frac{D_o}{D_e} \end{aligned}$$
  where $D_o$ is the observed disagreement and $D_e$ is the disagreement expected by chance^34^. It is commonly used in content analysis to quantify the extent of agreement between raters, and it differs from most other measures of inter-rater reliability because it calculates disagreement (as opposed to agreement). The key to adapting Alpha to multi-label data is in the definition of the disagreement function which specifies how different two annotations are. For nominal data, it is usually 0 for agreement and 1 for disagreement, but in multi-label tasks this binary notion is too coarse - two sets may partially overlap and should be considered “closer” than completely disjoint sets^35^. To allow for partial agreement between sets, Passonneau initially proposed the distance metric $d_P$ given by^36^:
  5 $$\begin{aligned} d_P(A,B) = {\left\{ \begin{array}{ll} 0, & \text {if } A = B \\ \frac{1}{3}, & \text {if } A \subset B \ \text {or} \ B \subset A \\ \frac{2}{3}, & \text {if } A \cap B \ne \emptyset , \ A \nsubseteq B, \ B \nsubseteq A \\ 1, & \text {if } A \cap B = \emptyset \end{array}\right. } \end{aligned}$$
  In later work, this approach was refined into the MASI (Measuring Agreement on Set-valued Items) distance^37^ by multiplying $d_P$ by the Jaccard distance $d_J$ (which is simply $1 -$ equation (3)):
  6 $$\begin{aligned} d_{\text {MASI}}(A,B) = d_P(A,B) \times \left( 1 - \frac{|A \cap B|}{|A \cup B|} \right) \end{aligned}$$
  This combination preserves the graded penalties for subset and intersection relations from $d_P$ while also scaling the penalty according to the proportion of overlap between sets. Using $d_{\text {MASI}}$ in the Alpha formula gives:
  7 $$\begin{aligned} \alpha _{\text {MASI}} = 1 - \frac{D_o^{\text {MASI}}}{D_e^{\text {MASI}}} \end{aligned}$$
  where $D_o^{\text {MASI}}$ and $D_e^{\text {MASI}}$ are computed with $d_{\text {MASI}}$ as the disagreement function^38^. This results in a chance-corrected reliability statistic that recognises full matches, subset relations, partial overlaps, and complete mismatches in a principled way. A similar approach using Jaccard Distance has been applied in multi-label annotation contexts^39^ and provides a more holistic view of inter-rater reliability when partial matches carry meaning.

**Note on Multi-Option Agreement Interpretation.** It is important to note that benchmarks for interpreting agreement coefficients are not universal, but depend strongly on the characteristics of the coding task, including the number of available labels and their empirical prevalence^34,35^. Agreement metrics such as Cohen’s $\kappa$ and Krippendorff’s $\alpha$ are therefore sensitive to category cardinality and label spartsity. This means that rigid cut-offs, such as the original benchmarks^40^, can be misleading in multi-category contexts^17^. Large-scale educational assessments illustrate this point: for instance, the NAEP scoring guidelines specify $\kappa> 0.70$ for two- or three-point items and $\kappa> 0.60$ for items with four to six categories, acknowledging that higher cardinality reduces attainable agreement^41^. To complement this reasoning, we conducted a task-specific simulation study that calibrates expected agreement values under random-like, near-disagreement, and near-perfect agreement scenarios. By matching the empirical distribution of label-set sizes observed in our data, the simulation provides interpretable upper and lower bounds for attainable agreement given the complexity of each multi-option task. We therefore interpret agreement relative to task difficulty rather than applying fixed universal thresholds. The description and results of the simulation can be found in Supplementary Material Section 5.

Additionally, we conducted a Monte Carlo simulation with 1000 bootstrap iterations, where for each iteration, we randomly resampled the coded ads with replacement and calculated agreement coefficients for the three dieticians and GPT using Gwet’s AC1 for single-option questions and Krippendorff’s Alpha for multi-option questions. We then determined whether GPT’s agreement coefficient fell within the range defined by the minimum and maximum human rater coefficients for that iteration. This generated bootstrap distributions of agreement coefficients for each rater across all question categories, allowing us to assess the percentage of simulations in which GPT’s performance was statistically indistinguishable from human expert variability.

### AI bias and other analyses

In order to evaluate systematic biases in AI model label selection compared to dietician or human consensus, we calculated option biases for each label by computing the mean difference in selection rates between humans and models using two-tailed z-tests. This measures the absolute tendency of each model to over- or under-select specific labels regardless of ad features. For *Alcohol*, *Ad Type*, *Target Group* and *WHO Categories*, we regard the dietician consensus as the domain benchmark, because of their expertise in nutrition and public health. Since *Premium Offers* and *Marketing Strategies* rely more on general interpretive judgement and are less nutrition-specific (also confirmed by the very low inter-dietician agreement for these two questions), we compare the AI labels to the crowd consensus instead.

Additionally, we have classified a smaller sample of 100 outdoor ads using the four AI models and the same three expert coders. The Ad Classification Task followed the same steps and instructions as in our main analysis. The only differences were the absence of the *text* of the ad, since the images represented for example pictures of outdoor billboards, and the addition of an extra question asking for any recognizable brands in the ad. The goal of this smaller analysis was to validate the ability of AIs to reliably classify food and alcohol advertisements, in a more noisy setting like outdoors. The results of the outdoor analysis are presented in Section 5 in Supplementary Material.

## Supplementary Information

Supplementary Information.

## Data Availability

The final datasets generated and analysed during the current study (sample of 1000 Meta ads and 100 outdoor ads, AI and human classifications) are available in the AI-validation Github repository, in the data folder, at https://github.com/Paularossi/AI-validation. The rest of the data are available from the corresponding author on request.

## Code availability

The respective Python and R code is stored on Github in the AI-validation repository, available at https://github.com/Paularossi/AI-validation.
