# Supplementary material for: Evaluating AI models for food and alcohol advertisement classification against human benchmarks
Source: Sci Rep. 2026 Mar 11;16:13058. doi: 10.1038/s41598-026-42426-x (PMC13099998; doi:10.1038/s41598-026-42426-x)
Supplement: Supplementary file 1 — Supplementary Information. [file 41598_2026_42426_MOESM1_ESM.pdf]

Evaluating AI Models for Food and Alcohol Advertisement Classification Against Human Benchmarks

Supplementary Material

Paula-Alexandra Gitu, Roberto Cerina, Alexander Grigoriev, Stefanie Vandevijvere

1. AI Classification

1.1 AI Classification Instructions

You will be provided with a picture of potential outdoor advertising in Belgium. You will be given sets of questions about various aspects of the advertisement along with definitions and examples. Please answer each question using the exact format: `*QUESTION LABEL*: Yes/No - Brief explanation`*. Do not include any extra text, greetings, or commentary.*

For example, your answers should look like: `*CARTOON*: Yes/No - explanation`*;* `*CELEBRITY*: Yes/No - explanation`*; and so on. Ensure that the question label is between a set of stars. Ensure that each answer includes a brief explanation of the features in the image that led to your choice. Ensure that you answer all questions.*

You will also be provided with a set of definitions which you should refer to when answering:

1. Food/drink manufacturing company or brand - a company or brand involved in producing and processing foods or beverages. Manufacturers focus on creating and packaging of consumable goods rather than selling directly to consumers. This category excludes restaurant/takeaway/delivery companies or brands and food retailers.

2. Food/drink retailer - a company or brand that sells food and drink products directly to consumers for home consumption (e.g., supermarkets, grocery stores, convenience stores and specialty food shops). These retailers primarily serve as intermediaries, providing a variety of products from different manufacturers to the end consumer. This category excludes manufacturing companies and restaurant/takeaway/delivery companies or brands.

3. Restaurant/takeaway/delivery outlet - a food service establishment that prepares and sells ready-to-eat meals and beverages for immediate consumption (either on the premises, through takeaway, or via delivery). These outlets focus on providing prepared food directly to customers for immediate or near-immediate consumption and do not primarily sell food or drink items in raw or packaged form for home cooking. This category excludes food retailers and manufacturing companies or brands.

4. Unprocessed or minimally processed food - natural foods (excluding Alcohol) that have undergone minimal changes (such as cleaning, drying, or freezing) without significant alteration to their nutritional content, e.g., fresh meat, eggs, frozen fruit.

5. Processed food - foods (excluding Alcohol) that have undergone processes like canning, smoking, fermentation or preservation, often with added ingredients to extend shelf life or enhance flavour, e.g., canned tomatoes, cheese, bread, smoked meat, dry fish.

6. Ultra-processed food - formulations of industrial ingredients (excluding Alcohol), resulting from a series of industrial processes such as frying, chemical modifications or application of additives, containing little or no whole foods, e.g., chips, candy, instant noodles, soft drinks, fast-food.

7. Processed culinary ingredients - substances (excluding Alcohol) extracted or refined from minimally processed foods, typically used in cooking or seasoning other food, e.g., sugar, butter, oils, spices.

List all distinct food items/dishes (not ingredients) observable in the image based on the overall presentation. Important: When the image features a composite food item (e.g., a burger, sandwich, or pizza), select only the single food category that best represents the whole item rather than listing separate categories for its individual ingredients (such as bread, meat, or sauces).

For each [of the 23] food category, answer in the following format: `*QUESTION LABEL*: Yes/No - Brief explanation`. If you answer '`Yes`' for a category, immediately provide a follow-up line indicating the level of processing for that food item in the format: `*<CATEGORY LABEL> PROCESSING*: <Processing Level> - Brief explanation`. Do not include any additional commentary. For example: `*CHOCOLATE SUGAR*: Yes - explanation`; `*CHOCOLATE SUGAR PROCESSING*: ULTRA PROCESSED - explanation`.

2. Qualtrics Survey

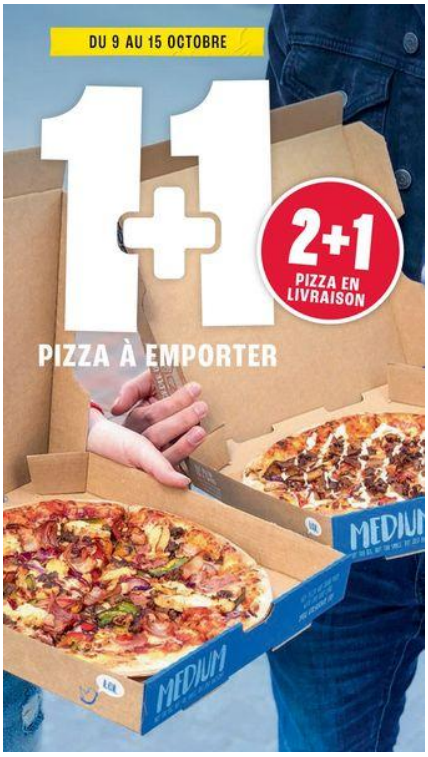

Figure S1 - Example of a Meta ad.

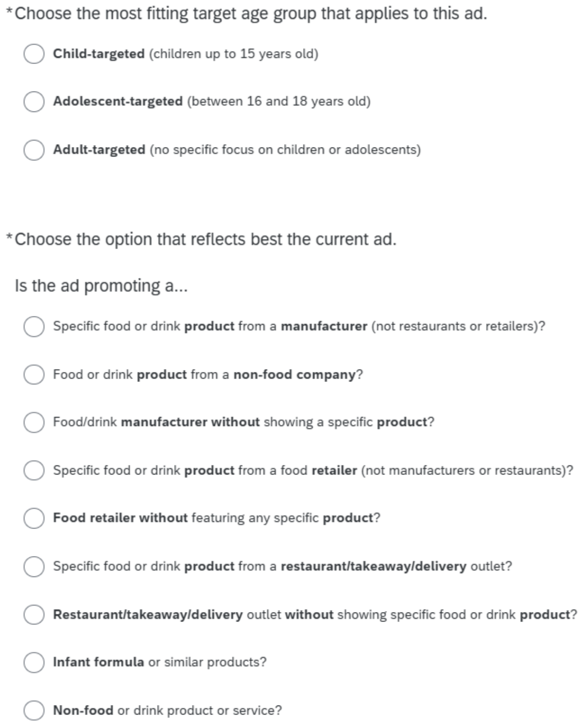

*Choose the most fitting target age group that applies to this ad.

- **Child-targeted** (children up to 15 years old)
- **Adolescent-targeted** (between 16 and 18 years old)
- **Adult-targeted** (no specific focus on children or adolescents)

*Choose the option that reflects best the current ad.

Is the ad promoting a...

- Specific food or drink **product** from a **manufacturer** (not restaurants or retailers)?
- Food or drink **product** from a **non-food company**?
- Food/drink **manufacturer without** showing a specific **product**?
- Specific food or drink **product** from a food **retailer** (not manufacturers or restaurants)?
- **Food retailer without** featuring any specific **product**?
- Specific food or drink **product** from a **restaurant/takeaway/delivery** outlet?
- **Restaurant/takeaway/delivery** outlet **without** showing specific food or drink **product**?
- **Infant formula** or similar products?
- **Non-food** or drink product or service?

Figure S2 - Questions example from the Qualtrics survey.

3. AI Bias Analysis

This section presents the results of the AI Bias Analysis for the remaining single-option questions: Alcohol, Target Group and Ad Type.

Panel A (Alcohol) shows minimal bias across all models, with values close to zero. Panel B (Target Group) shows consistent significant under-detection of the *Adult* group across all models (ranging from -0.10 to -0.15), and an over-detection of *Child* content. The latter suggests that AI models are more likely than humans to identify ads as child-targeted. Panel C (Ad Type) shows more complex bias patterns, with most notable negative bias across all models for Manufacturer without a Product (from -0.06 to -0.15) and Restaurant without a Product (from -0.08 to -0.10). Slight over-detection was observed for Restaurant with Product, but overall it seems the models struggled with identifying ads that contained no products.

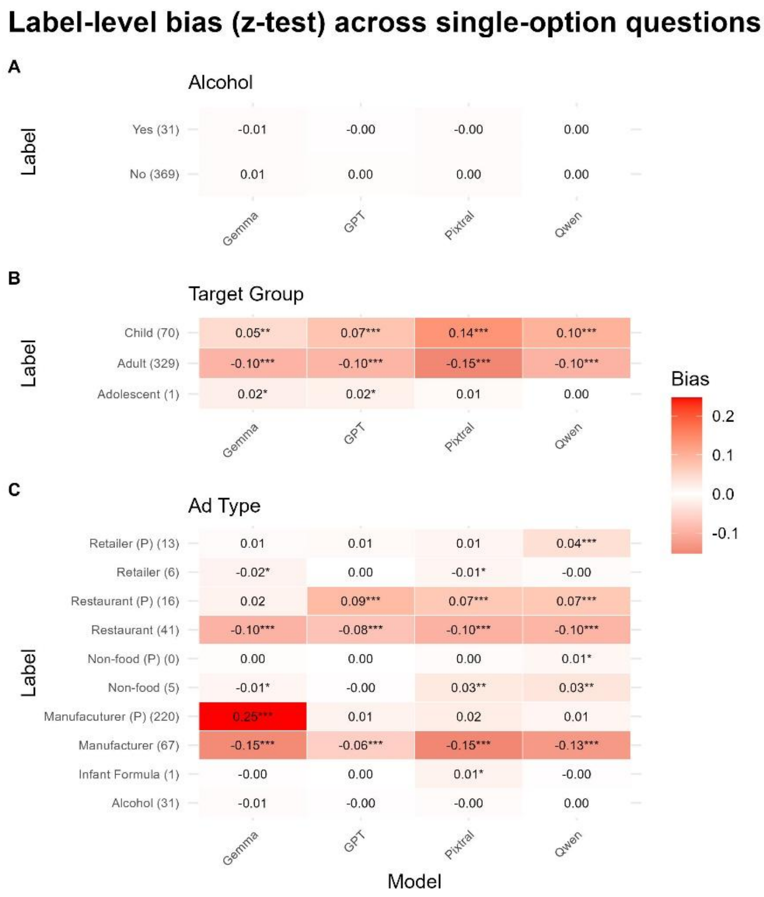

*Figure S3 - Label-level bias in AI model predictions for single-option questions compared to dietician consensus for **Panel A**: Alcohol, **Panel B**: Target Group and **Panel C**: Ad Type. The cells show option bias (mean selection rate difference: model - human) with z-test significance (*p < 0.05, **p < 0.01, ***p < 0.001). Positive values indicate over-selection; negative values indicate under-selection. The numbers in the brackets next to each label represent the number of ads in which the label was selected by the dietician consensus out of the total sample of 400 ads.*

4. Language Analysis

We compare agreement separately by language to examine whether certain classification tasks are more sensitive to linguistic context. To keep the analysis focused, we restrict attention to the two strongest-performing models, GPT and Qwen, to highlight the most relevant cross-language differences. Additionally, we control for Dutch- and French-only ads, which account for the largest and most comparable language groups in the data and represent primary languages used in public health advertising research in Belgium. English and bilingual ads were not included due to their smaller sample size and heterogeneous communicative intent. Figure S4 below presents the delta in agreement for the four questions that showed most variation. The results show that for Marketing Strategies, both GPT and Qwen generally aligned more closely with dieticians and consensus in Dutch ads, with differences up to $\Delta \approx +0.10$. For WHO Categories, agreement was markedly higher in

Dutch ads, especially for GPT and Qwen against the crowd consensus (Δ ≈ +0.12). Although the overall pattern points to Dutch ads being slightly easier for both GPT and Qwen to classify in multi-option settings, these differences were not statistically significant and insufficient to draw meaningful conclusions about language-specific variations. Exact sample sizes, per-language agreement estimates and confidence intervals for all reported comparisons are provided in Table S1.

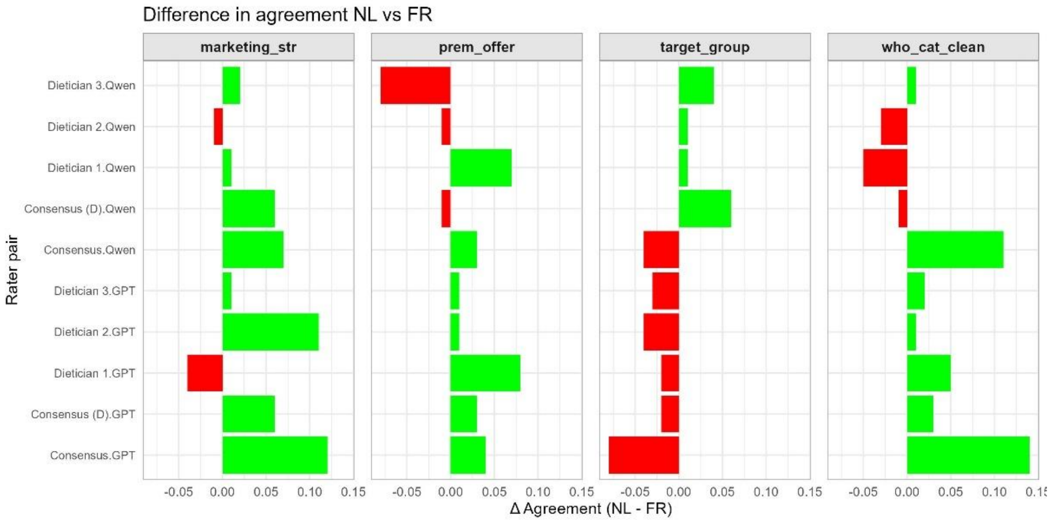

Figure S4 - Difference in agreement (Δ Krippendorff's Alpha for Marketing Strategies, Premium Offers and WHO Categories and Δ Gwet's AC1 for Target Group) between Dutch and French ads for GPT and Qwen compared to dieticians and consensus labels, across different tasks. Positive values indicate higher agreement in Dutch, negative values indicate higher agreement in French. None of the differences were statistically significant.

Question	Language	n	Pair	Kripp. α	95% CI	p
Marketing Strategies	NL	149	GPT-Cons	0.53	(0.421, 0.630)	0.14
	FR	125	GPT-Cons	0.40	(0.278, 0.530)	
	NL	149	Qwen-Cons	0.39	(0.288, 0.490)	0.35
	FR	125	Qwen-Cons	0.31	(0.197, 0.432)	
	NL	149	GPT-Cons D.	0.44	(0.339, 0.547)	0.46
	FR	125	GPT-Cons D.	0.38	(0.264, 0.503)	
	NL	149	Qwen-Cons D.	0.41	(0.318, 0.499)	0.41
	FR	125	Qwen-Cons D.	0.35	(0.239, 0.460)	
Premium Offers	NL	149	GPT-Cons	0.64	(0.537, 0.751)	0.54
	FR	125	GPT-Cons	0.60	(0.503, 0.696)	
	NL	149	Qwen-Cons	0.57	(0.477, 0.671)	0.61
	FR	125	Qwen-Cons	0.54	(0.450, 0.629)	
	NL	149	GPT-Cons D.	0.59	(0.483, 0.702)	0.68
	FR	125	GPT-Cons D.	0.56	(0.453, 0.667)	
	NL	149	Qwen-Cons D.	0.46	(0.363, 0.565)	0.88
	FR	125	Qwen-Cons D.	0.47	(0.380, 0.570)	
Target Group	NL	149	GPT-Cons	0.80	(0.724, 0.881)	0.12
	FR	125	GPT-Cons	0.88	(0.819, 0.949)	
	NL	149	Qwen-Cons	0.81	(0.737, 0.889)	0.44
	FR	125	Qwen-Cons	0.85	(0.782, 0.927)	

	NL	149	GPT-Cons D.	0.90	(0.842, 0.956)	0.69
	FR	125	GPT-Cons D.	0.91	(0.860, 0.970)	
	NL	149	Qwen-Cons D.	0.92	(0.866, 0.968)	0.26
	FR	125	Qwen-Cons D.	0.86	(0.779, 0.943)	
WHO Food Categories	NL	149	GPT-Cons	0.47	(0.396, 0.550)	0.01
	FR	125	GPT-Cons	0.33	(0.253, 0.415)	
	NL	149	Qwen-Cons	0.46	(0.380, 0.533)	0.07
	FR	125	Qwen-Cons	0.35	(0.265, 0.434)	
	NL	149	GPT-Cons D.	0.50	(0.430, 0.580)	0.58
	FR	125	GPT-Cons D.	0.47	(0.390, 0.557)	
	NL	149	Qwen-Cons D.	0.45	(0.372, 0.529)	0.91
	FR	125	Qwen-Cons D.	0.46	(0.371, 0.543)	

Table S1 - Agreement between GPT and Qwen and the human reference standard separately for Dutch (NL) and French (FR) ads. For each question, the number of ads per language (n), the agreement between a rater pair (Gwet's AC1 for Target Group and Krippendorff's α with MASI distance for the others), and corresponding 95% confidence intervals are shown. P-values refer to two-sided tests of differences in agreement between NL and FR ads.

5. Simulation Study for Interpreting Multi-Label Agreement

To contextualize agreement magnitudes in complex multi-label annotation tasks, we conducted a simulation study based on a probabilistic annotation-noise model inspired by Raykar et al. (2010). Each annotator is represented as a noisy observer of an underlying ground truth, with sensitivity (true-positive rate, TPR) and specificity (false-positive rate, FPR) used as parameters. By using the same inclusion and exclusion probabilities independently at the label level, we extend this method to set-valued annotations. This abstraction maintains interpretability and aligns with known models of annotation noise, while allowing us to calibrate expected agreement values under controlled levels of underlying alignment.

Latent label-set sizes are sampled from the empirical distribution observed in the annotation data for each question, ensuring that simulated tasks reflect the sparsity and structure induced by the questionnaire design (see Table S2 below for empirical sizes).

Question	***# Labels***	***Mean***	***Median***	***Empty Prop.***	***Max Size***
Premium Offers	10	1.10	1	0.0073	9
Marketing Strategies	11	1.07	1	0.0073	6
WHO Categories	26	1.33	1	0.0057	23

*Table S2 - Empirical label-set sizes per question. Each row summarizes 3000 coder annotations (3 coders * 1000 ads), reporting the mean, median, proportion of empty responses, and maximum number of labels assigned for one ad, which are used in the latent-set generator in the simulation.*

Three simulation scenarios were considered to represent different levels of latent alignment between annotators and an underlying ground truth. These scenarios are parameterized by the TPR and FPR at the label level, and are shown in Table S3.

Scenario	*TPR*	*FPR*	*Interpretation*
Near-disagreement	0.3	0.1	Low alignment with ground truth
Random-like	0.5	0.05	Moderate alignment
Near-perfect	0.9	0.01	High alignment

Table S3 - Description and parameters for the three simulated agreement scenarios.

The pseudocode for the simulation is presented below:

Inputs

- $N = 1000$: number of items (ads)
- $R = 3$: number of coders per item
- K: number of possible labels for the question
- S: empirical distribution of observed label-set sizes
- TPR, FPR: annotation-noise parameters (Table S3)

Output

- $S_{i,r}$: simulated label set for item i and coder r

```
FOR each item i = 1 to N:
    # Step 1: sample latent true label set size
    s_i <- sample from empirical distribution S
    s_i <- clamp(s_i, 0, K) # ensure label set size is set between 0 and K

    # Step 2: generate latent true label set
    IF s_i = 0:
        T_i <- Ø
    ELSE:
        T_i <- sample s_i labels uniformly from {1,…,K} without replacement

    # Step 3: generate coder annotations
    FOR each coder r = 1 to R:
        S_i,r <- Ø
        FOR each label k in {1,…,K}:
            IF k ∈ T_i:
                include k in S_i,r with probability TPR
            ELSE:
                include k in S_i,r with probability FPR
        END FOR
    END FOR
END FOR
```

For each simulated item (ad), the size of the latent label set is first sampled from the empirical distribution of observed set sizes in our original data (in the $[0, K]$ range). Given the sampled size, the “true” label set is generated by selecting labels uniformly at random. Each coder’s annotation is then generated independently by including labels from the true set with probability equal to the TPR and including labels not in the true set with probability FPR (varies by scenario).

After the simulation, Jaccard similarity and Krippendorff’s α with MASI distance are computed for each coder pair. With three coders per item, the final agreement estimate is obtained by averaging results over the three coder pairs. Table S4 reports the expected agreement metrics under each simulation scenario, averaged across coder pairs.

Question	*Scenario*	*Avg. α*	*Avg. Jaccard*
**Premium Offers**	Near-disagreement	0.03	0.16
	Random-like	0.15	0.30
	Near-perfect	0.69	0.78
**Marketing Strategies**	Near-disagreement	0.01	0.13
	Random-like	0.14	0.28
	Near-perfect	0.67	0.77
**WHO Categories**	Near-disagreement	0.01	0.08
	Random-like	0.06	0.16
	Near-perfect	0.60	0.70

Table S4 - Average Jaccard similarity and Krippendorff's α (MASI distance) computed from simulated multi-label annotations for each question and scenario, with empirically matched label-set sizes. Agreement values are averaged across the three coder pairs and serve as task-specific benchmarks for interpreting empirical agreement magnitudes.

Under the near-perfect agreement scenario, average Krippendorff's α across coder pairs ranged from 0.60 to 0.69 across the three questions, reflecting the difficulty of achieving high agreement in complex multi-option annotation tasks. For context, the α values observed in the main analysis (e.g., $\alpha \approx 0.45$ for WHO Categories) lie well above the values obtained under the random-like scenario and below the optimistic upper bound defined by the near-perfect simulation, indicating substantial alignment given the size and sparsity of the label space.

Even under high latent alignment, agreement coefficients remain attenuated due to the combinatorial penalties inherent to set-valued annotations. Accordingly, simulated agreement values should be interpreted as task-specific benchmarks indicating what levels of agreement are reasonable given the complexity of the annotation problem.

6. Outdoor Ads

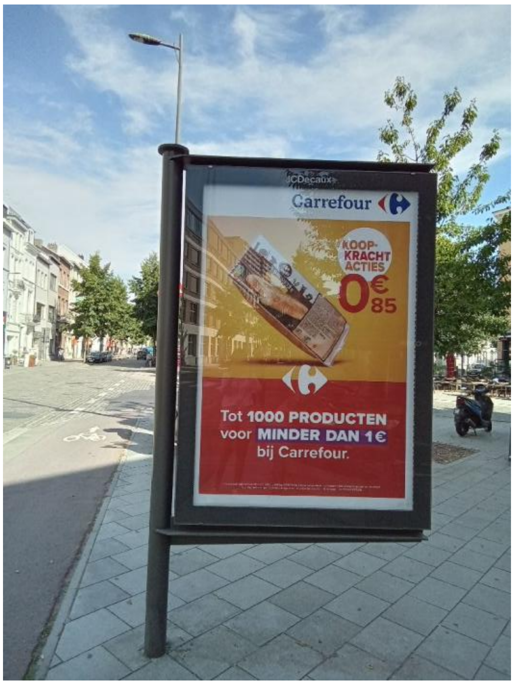

Figure S5 – Example of an outdoor ad

For outdoor advertisements, all four models achieved high agreement with dietician consensus across all single-option questions, as seen in Figure S6. Agreement was nearly perfect for Alcohol (0.97-0.99) and remained strong for Ad Type (0.69-0.80) and Target Group (0.85-0.96), the latter two showing an even higher agreement than for the Meta ads. These values were well within the range of inter-dietician reliability, confirming that the AI models performed comparably to dieticians even in less-structured outdoor imagery.

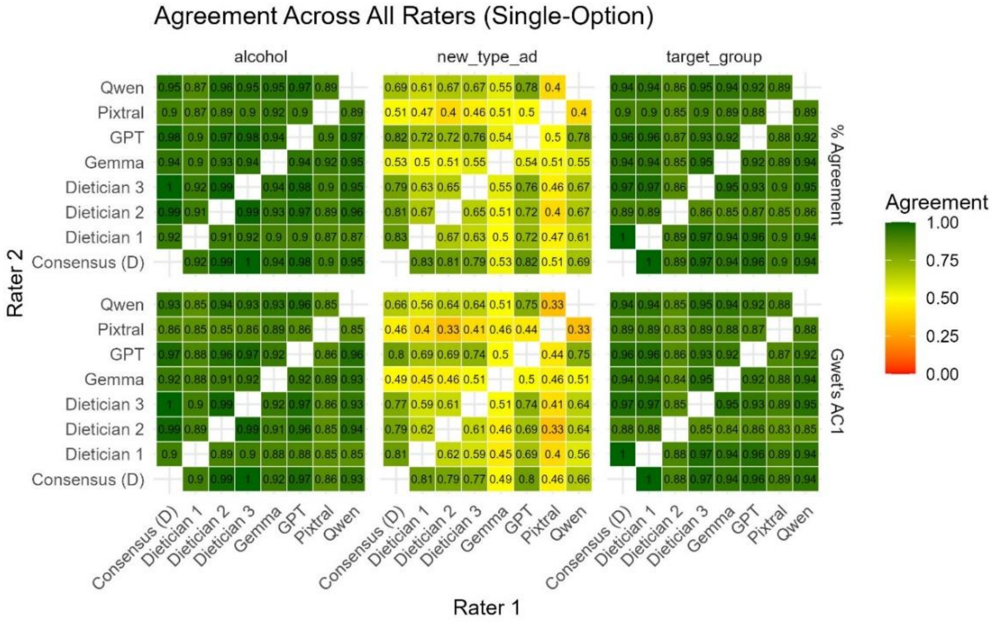

Figure S6 - Pairwise agreement between dieticians and the AI models for single-option variables. Values represent both proportion agreement (top) and Gwet's AC1 (bottom). The agreement is computed over the full set of 100 outdoor ads.

As in the main dataset, agreement levels were lower for multi-option variables, as shown in Figure S7. Jaccard similarities typically ranged from 0.55 to 0.84 for Premium Offers and Marketing Strategies, and from 0.5 to 0.9 for WHO Food Categories, with lower corresponding Krippendorff's α values. In this setting, Pixtral performed considerably worse than the other models, producing negative agreement values for Premium Offers. While this indicates moderate to low alignment, GPT's performance remained comparable to or slightly above inter-dietician agreement, suggesting robustness across both online and outdoor advertising contexts.

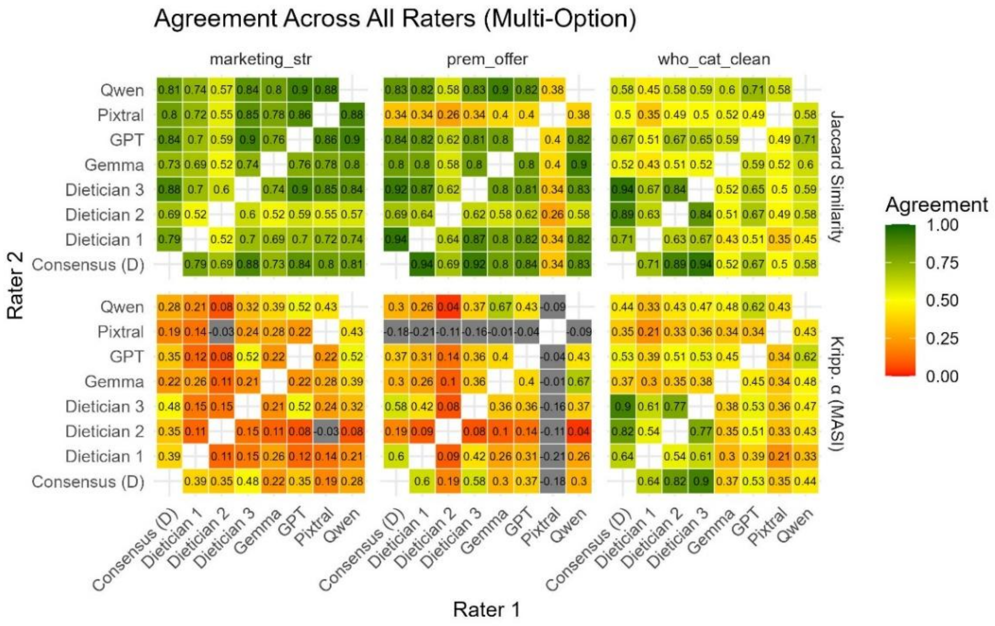

Figure S7 - Pairwise agreement between dieticians and the AI models for multi-option variables. Values represent Jaccard similarity (top) and Krippendorff's Alpha with MASI distance (bottom). The agreement is computed over the full set of 100 outdoor ads.

For the additional Brand question included in the outdoor ad analysis, GPT reached a Jaccard similarity of 0.71-0.78 and a Krippendorff's α between 0.69-0.76 when compared to dietician consensus, outperforming all the other models by a considerable margin. These values closely matched inter-dietician agreement, indicating that GPT was similarly reliable in identifying brand presence or logos across diverse outdoor ad contexts.

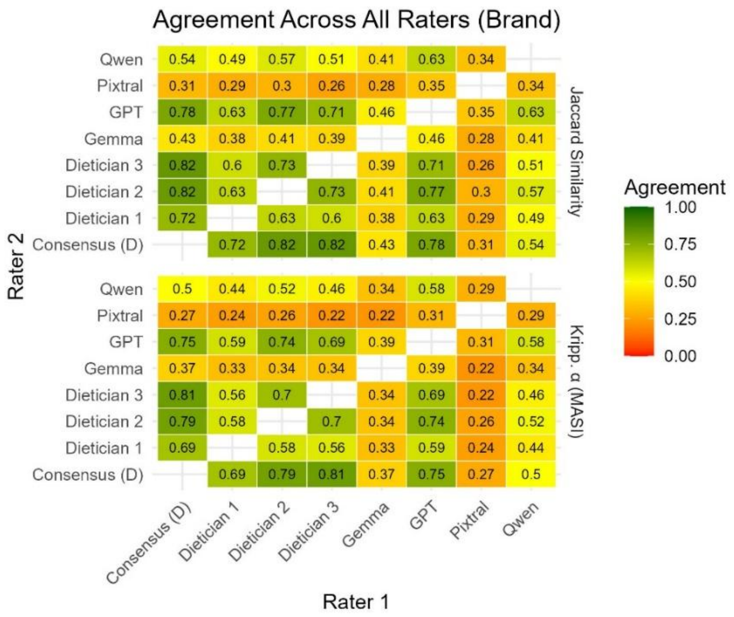

Figure S8 - Pairwise agreement between dieticians and GPT for the brand question. Values represent Jaccard similarity (top) and Krippendorff's Alpha with MASI distance (bottom). The agreement is computed over the full set of 100 outdoor ads.

7. Sensitivity Analysis of Multi-Label Consensus Rules

For multi-option annotation tasks (e.g., Premium Offers or WHO Food Categories), the construction of consensus labels is a critical methodological step that can influence agreement estimates. Unlike single-option variables, multi-option questions allow annotators to select multiple labels simultaneously, increasing the likelihood of partial overlap and disagreement. As a result, different consensus rules may trade off strictness against coverage, potentially affecting both agreement magnitudes and model rankings.

In the main analysis, consensus labels for multi-option variable were constructed using the following *threshold-plus-union rule*: a label was included in the consensus if at least two out of three coders selected it. If not label met this threshold for a given ad, the consensus was defined as the union of all labels selected by any coder. This fallback was chosen to avoid assigning empty consensus labels in cases where coders identified different, but potentially relevant features.

To assess the practical impact of this fallback, we quantified how frequently it was triggered during the consensus construction, presented in Table S5 below.

**Question**	*% fallback for consensus of*	
	**Dieticians (400)**	**Crowd (1000)**
Marketing Strategies	15.2%	13.1%
Premium Offers	4.5%	5.2%
WHO Categories	3.5%	9%

Table S5 - Percentage of ads for which the union fallback was triggered during consensus construction for multi-option questions, reported separately for dietician-based (for the subsample of 400 ads) and crowd-based consensus (for the full sample of 1000 ads).

To evaluate whether agreement outcomes were sensitive to this design choice, we conducted a sensitivity analysis comparing the original consensus rule to three alternative constructions:

1. **Threshold-only** consensus (≥2 of 3; empty fallback): labels are included only if selected *by at least two coders* (like in the original rule); otherwise consensus is empty (instead of taking the union).
2. **Union-only** consensus: the consensus includes all labels selected *by at least one coder*, irrespective of agreement.
3. **Intersection-only** consensus: the consensus includes only labels selected *by all three coders*.

These alternatives range from permissive (union-only) to very conservative (intersection-only). Figure S9 (dieticians) and Figure S10 (crowd) show the sensitivity of agreement outcomes (Krippendorff's α) to these alternative consensus rules, relative to the original *threshold-plus-union* rule.

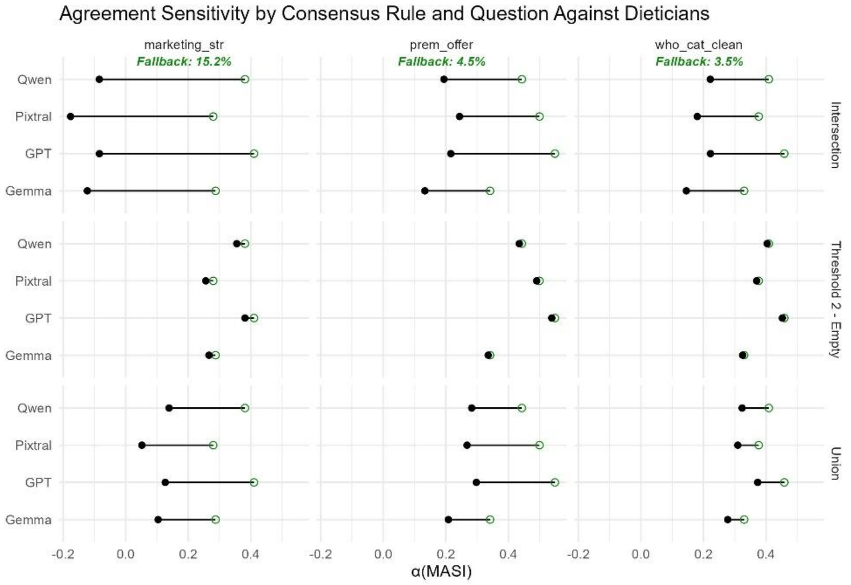

Figure S9 - Agreement sensitivity between AI models and dietician consensus to alternative multi-label consensus rules. Green open circles indicate agreement under the original threshold-plus-union rule and are constant within each question by model. Black filled circles show agreement obtained under alternative consensus rules, connected by horizontal lines to the agreement under the original baseline. Agreement is measured using Krippendorff's α and fallback percentages indicate how often the original union fallback was triggered for each question.

Across all questions and models, alternative consensus rules result in lower agreement values, as expected from stricter definitions of consensus. The *threshold-only* rule with an empty fallback produces the smallest deviation from the original rule, suggesting that adding a union fallback has minimal impact on agreement estimates. More permissive (*union-only*)

and more conservative (*intersection-only*) rules yield larger changes, but the relative ordering of models remains unchanged. These results are consistent across

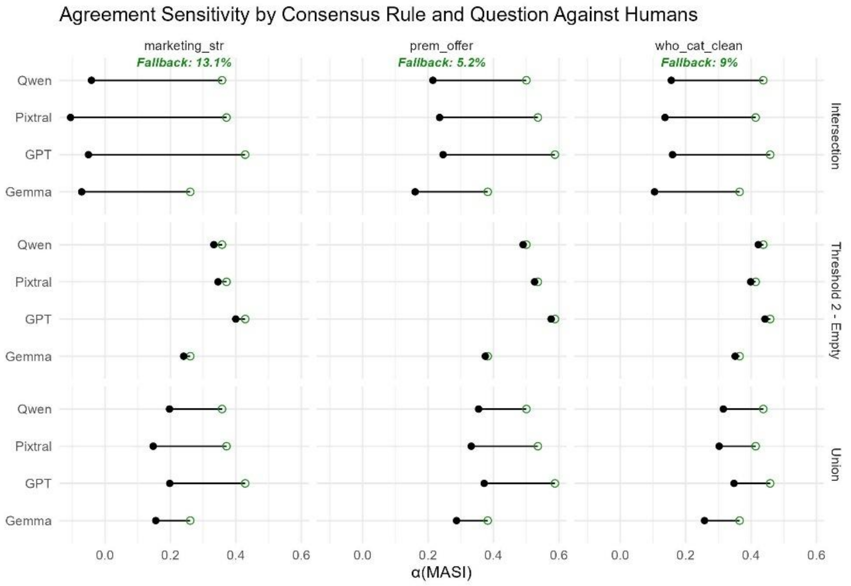

Figure S10 - Agreement sensitivity between AI models and crowd consensus to alternative multi-label consensus rules. Green open circles indicate agreement under the original threshold-plus-union rule and are constant within each question by model. Black filled circles show agreement obtained under alternative consensus rules, connected by horizontal lines to the agreement under the original baseline. Agreement is measured using Krippendorff's α and fallback percentages indicate how often the original union fallback was triggered for each question.

Taken together, the results of the sensitivity analysis show that while absolute agreement levels depend on the strictness of the consensus definition, the use of a union fallback in the original rule does not meaningfully inflate agreement. The main conclusions of the analysis are therefore robust to variations in multi-label consensus construction.

8. Bootstrap Uncertainty Estimates

Question	Median agreement	95% CI	% within human range
**Alcohol**	1.00	(0.99, 1.00)	53.6
**Target Group**	0.89	(0.85, 0.92)	99.4
**Ad Type**	0.72	(0.68, 0.77)	55.9
**Premium Offers**	0.62	(0.57, 0.66)	97.7
**Marketing Strategies**	0.62	(0.57, 0.66)	97.5
**WHO Food Categories**	0.78	(0.74, 0.82)	95.1

Table S6 - Bootstrap-based uncertainty estimates of GPT's agreement relative to dietician consensus. For each question, the table shows the median agreement, 95% bootstrap confidence interval across 1000 Monte Carlo resamples, and the percentage of simulations in which GPT's agreement falls within the empirically observed range of dietician agreement. Single-option questions use Gwet's AC1, while multi-option questions use Krippendorff's a with MASI distance.

References

Raykar, V. C., Yu, S., Zhao, L. H., Valadez, G. H., Florin, C., Bogoni, L., & Moy, L. (2010). Learning From Crowds. *Journal of Machine Learning Research, 11*(43), 1297-1322. Retrieved from http://jmlr.org/papers/v11/raykar10a.html
